# Combined multiphoton imaging and biaxial tissue extension for quantitative analysis of geometric fiber organization in human reticular dermis

**DOI:** 10.1038/s41598-019-47213-5

**Published:** 2019-07-23

**Authors:** Maho Ueda, Susumu Saito, Teruasa Murata, Tomoko Hirano, Ryoma Bise, Kenji Kabashima, Shigehiko Suzuki

**Affiliations:** 1 Department of Plastic and Reconstructive Surgery, Graduate School of Medicine and Faculty of Medicine, Kyoto University, Kyoto, Japan; 2 Department of Dermatology, Graduate School of Medicine and Faculty of Medicine, Kyoto University, Kyoto, Japan; 3 Department of Advanced Information Technology, Kyushu University, Fukuoka, Japan

**Keywords:** Anatomy, Imaging

## Abstract

The geometric organization of collagen fibers in human reticular dermis and its relationship to that of elastic fibers remain unclear. The tight packing and complex intertwining of dermal collagen fibers hinder accurate analysis of fiber orientation. We hypothesized that combined multiphoton microscopy and biaxial extension could overcome this issue. Continuous observation of fresh dermal sheets under biaxial extension revealed that the geometry of the elastic fiber network is maintained during expansion. Full-thickness human thigh skin samples were biaxially extended and cleared to visualize the entire reticular dermis. Throughout the dermis, collagen fibers straightened with increased inter-fiber spaces, making them more clearly identifiable after extension. The distribution of collagen fibers was evaluated with compilation of local orientation data. Two or three modes were confirmed in all superficial reticular layer samples. A high degree of local similarities in the direction of collagen and elastic fibers was observed. More than 80% of fibers had directional differences of ≤15°, regardless of layer. Understanding the geometric organization of fibers in the reticular dermis improves the understanding of mechanisms underlying the pliability of human skin. Combined multiphoton imaging and biaxial extension provides a research tool for studying the fibrous microarchitecture of the skin.

## Introduction

Currently, the mechanisms underlying the pliability of human skin are unclear. Human skin is composed of the epidermis, dermis, and hypodermis. The dermis is divided into a more superficial layer called the papillary dermis and a deeper layer called the reticular dermis. The maintenance of the reticular dermis is closely associated with good functional results after skin grafting. For example, full-thickness skin grafts exhibit less contracture during the postoperative healing process than split-thickness skin grafts^1^. Full-thickness skin grafts are believed to provide better texture and softness than split-thickness skin grafts^2^ and are preferentially used in the reconstruction of sites with high functional importance such as the face, neck, and joints in the extremities^3^. This implies that the reticular dermis is involved in imparting pliability to the skin. The dermis consists of fibrous components including collagen fibers, elastic fibers, and ground substances. Collagen fibers in the dermis are composed of type I and type III collagen^4^. In the reticular dermis, relatively large collagen fibers with wavy morphology form densely packed and complexly intertwining structures with horizontal laminar organization^4–7^. The structure of the dermis varies by depth. For example, collagen fibers are thicker in the deep reticular dermis and reportedly are the densest in the middle dermal zone^4,7^. The diameter of elastic fibers also varies by depth^8^. Mechanically, the extensibility of the skin is derived not only from the straightening of wavy collagen fibers but also from the deformation of their higher order structure^9–12^. The deformability of fibrous structures depends on their geometric organization. Therefore, for more complete understanding of the mechanisms underlying skin pliability, it is crucial to know the geometric organization of collagen fibers in the reticular dermis and how it varies by depth.

The geometric organization of the human dermis has been a subject of controversy. Many histological studies have revealed that collagen fibers have a preferred orientation^13–15^. However, scanning electron microscopy studies failed to support this finding. Instead, they suggest that the network of collagen fibers is basically random^5,6^. The lack of standardized methods by which the geometric organization of the dermis has been assessed quantitatively and three-dimensionally is a fundamental issue underlying this controversy. Moreover, there is little information on how the structure of elastic fibers and collagen fibers are related, even though the recoiling force of elastic fibers is believed to be the force that leads to contraction of extended collagen fibers^11^.

In order to quantitatively analyze the geometric organization of collagen fibers in the entire reticular dermis and its relationship to the geometric organization of elastic fibers, clear, high-resolution, three-dimensional visualization of these fibers is essential. Multiphoton microscopy (MPM) is an emerging imaging method based on non-linear optical effects produced by near-infrared femtosecond lasers^16,17^. Second harmonic generation (SHG) is an MPM imaging mode that helps visualize intra-organ collagen without labeling them^18^. Two-photon excited autofluorescence (TPAF) is another MPM imaging mode. TPAF signals from elastic fibers allow for imaging without labeling^19,20^. Since labeling is unnecessary, architectural changes in fibrous networks during tissue processing can be avoided. In contrast, attenuation of the laser beam between the papillary dermis and reticular dermis results in incomplete visualization of the reticular dermis^21^. In addition, the complex entangled and tightly packed structure of collagen fibers fundamentally precludes the identification of fiber continuity and results in incomplete evaluation of fiber orientation. If the orientation of collagen fibers cannot be identified, the directional relationship between collagen and elastic fibers cannot be evaluated quantitatively.

This study aimed to characterize the geometric organization of collagen fibers in the entire human reticular dermis layer *ex vivo* using a new microscopic technique that combines MPM, the tissue clearing method, and biaxial extension. We hypothesized that tissue clearing would allow observation of deep dermal layers and biaxial extension would facilitate identification of individual collagen fibers, so the combination would allow for quantitative analysis of fiber orientation. Specifically, the study focused on architectural differences between the superficial and deep layers of the reticular dermis and the distributional relationship between collagen and elastic fibers.

## Results

### Determination of the degree of extension for microscopic observation

The degree of biaxial extension in skin samples was determined based on mechanical tests. Pairs of dermal strips orthogonal to each other were used to obtain a stress–strain curve. The stress–strain curve for human skin is characterized by a low-stress region followed by a high-stress region^10,14^. In the low stress region, undulated collagen fibers became linear^9,10^; the stretch ratio in this region was similar whether parallel or perpendicular to the direction of body hair (1.28 ± 0.06 vs. 1.26 ± 0.07; n = 6). Consequently, the degree of biaxial extension before the clearing process was set to 1.25.

### Characterization of architectural changes in the fiber network of the reticular dermis with biaxial extension

**Figure 1:**
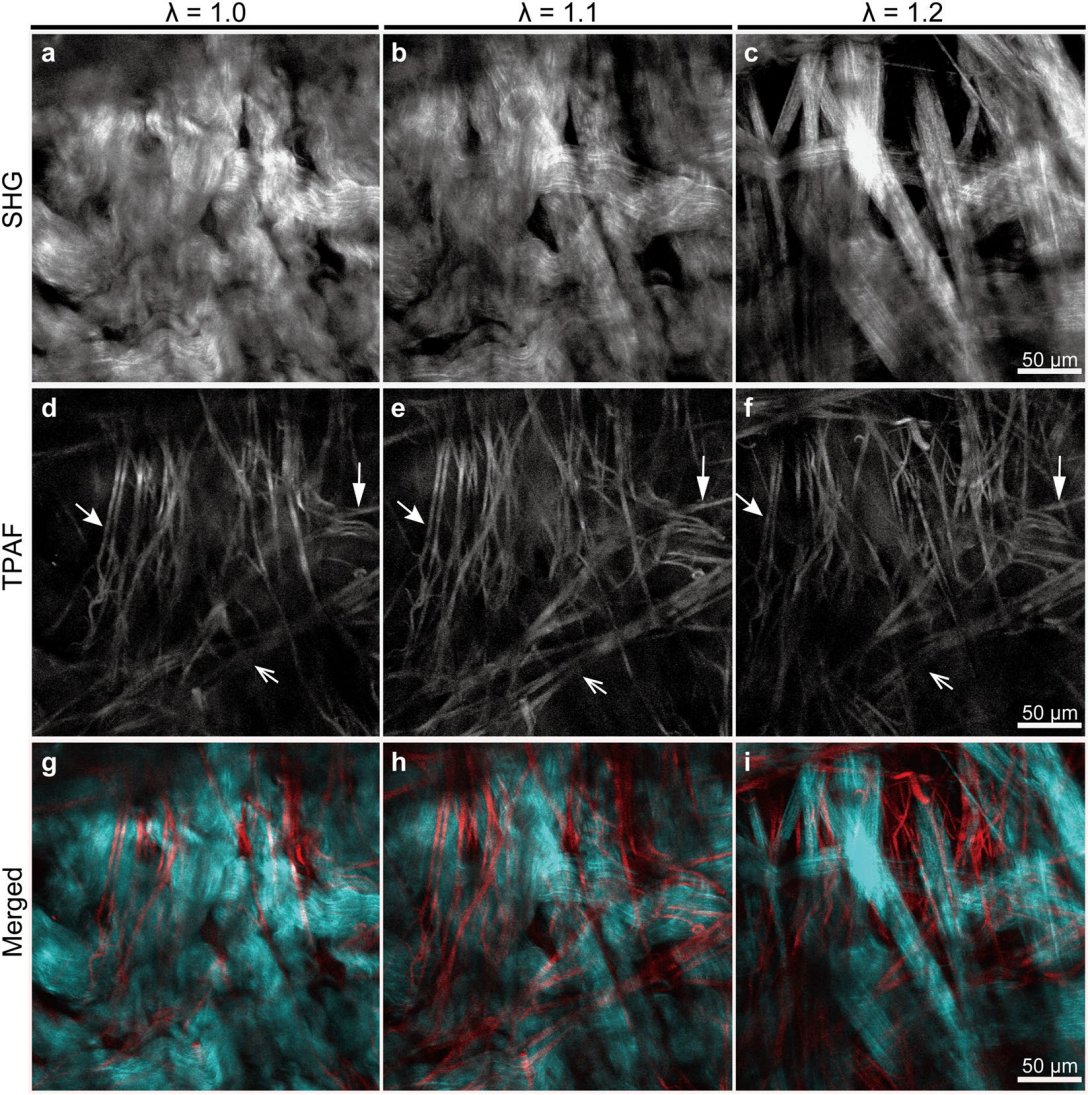
Dynamic multiphoton microscopy images of dermal fiber architecture under biaxial tissue extension. Second harmonic generation (SHG) images show architectural changes in collagen fibers (**a**–**c**) whereas two-photon autofluorescence (TPAF) images show architectural changes in elastic fibers (**d**–**f**). λ represents the stretch ratio. Arrows of the same shape indicate the same elastic fiber. Note that in TPAF images, the direction and shape of elastic fibers were maintained when stretched, whereas collagen fibers became linear with extension.

**Figure 2:**
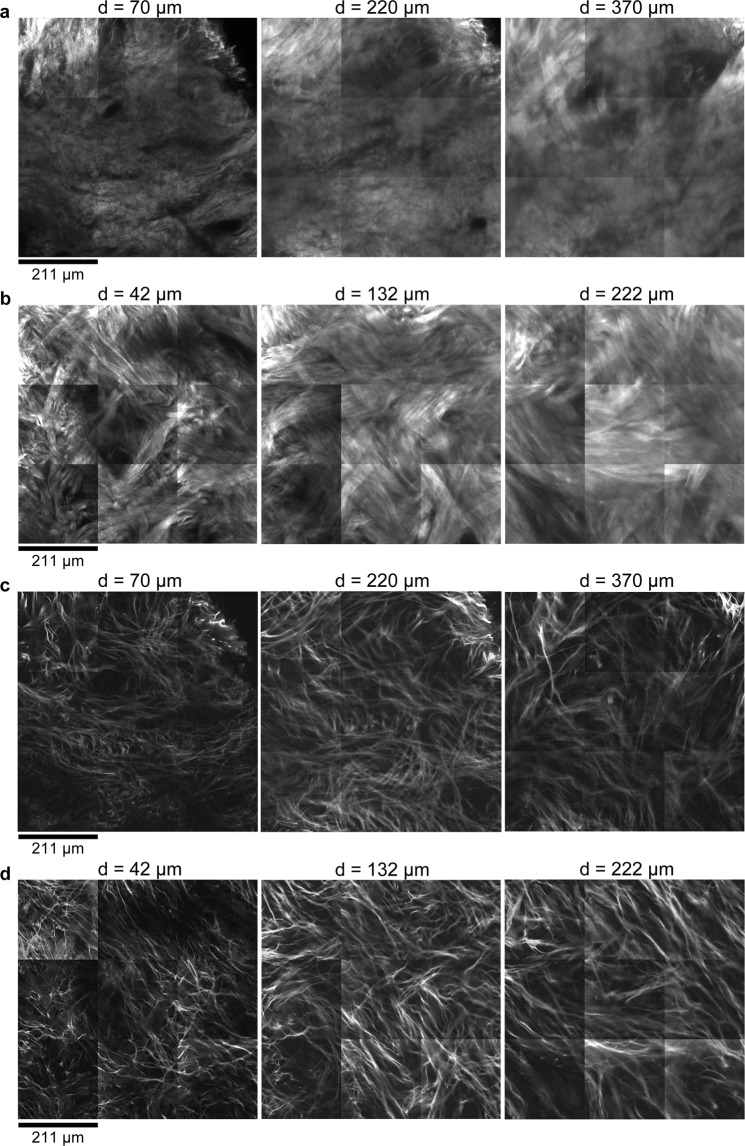
Impact of biaxial extension on the visibility of collagen architecture in the reticular dermis. Multiphoton microscopy images of the reticular dermis in unstretched (**a**,**c**) and biaxially extended (**b**,**d**) human skin samples. (**a**,**b**), Second harmonic generation (SHG) images. (**c**,**d**) Two-photon autofluorescence images. d indicates the distance from the papillary dermis–reticular dermis boundary. Since the skin was thinned 0.6-fold due to biaxial extension, the images in (**b**,**d**) are from 0.6 times the depth of the images in (**a**,**c**) for comparison.

To verify whether the original geometric organization of collagen and elastic fibers is conserved during biaxial extension, dynamic microscopic observation was performed. Using fresh human dermal sheets, a dynamic analysis of architectural changes in fibers within the reticular dermis under biaxial extension was conducted. In the resting state, collagen fibers were tightly packed; thus, assessing the individual distribution of these fibers was difficult. However, this tight packing of fibers decreased with tissue extension, which increased the gap between fibers. Ultimately, fibers could be observed in a linear form (Fig. 1a–c). Similarly, elastic fibers expanded while retaining the initial mesh-like architecture (Fig. 1d–f). Directional similarities in the major component vectors of elastic fibers before and after extension evaluated based on cosine similarity revealed a high degree of similarity (degree of similarity > 0.99; n = 2), suggesting that the elastic fiber network similarly expands with biaxial extension. In addition, during the latter half of extension, collagen fibers retained the same direction (Fig. 1b,c). Next, the impact of biaxial extension on the visibility of collagen fibers in full-thickness skin samples was evaluated. Pairs of skin samples (n = 3) with and without biaxial extension were fixed and cleared. The reticular dermis was observed using MPM. Stacked SHG images clearly showed the three-dimensional architecture of collagen fibers under biaxial extension (Fig. 2). Based on the preliminary experimental data, in which skin thickness after biaxial extension was on average 0.6 times the thickness before biaxial extension, images of the stretched samples were observed at 0.6 times the depth used to observe the unstretched samples. In unstretched skin samples, identification of an individual fiber was difficult across all layers of reticular dermis collagen fibers. In contrast, in skin samples under biaxial extension, fibers with linear morphology and increased inter-fiber clearances were observed, allowing for identification of fiber continuity.

### Morphological characteristics of collagen and elastic fibers and the local relationship of their orientations

**Figure 3:**
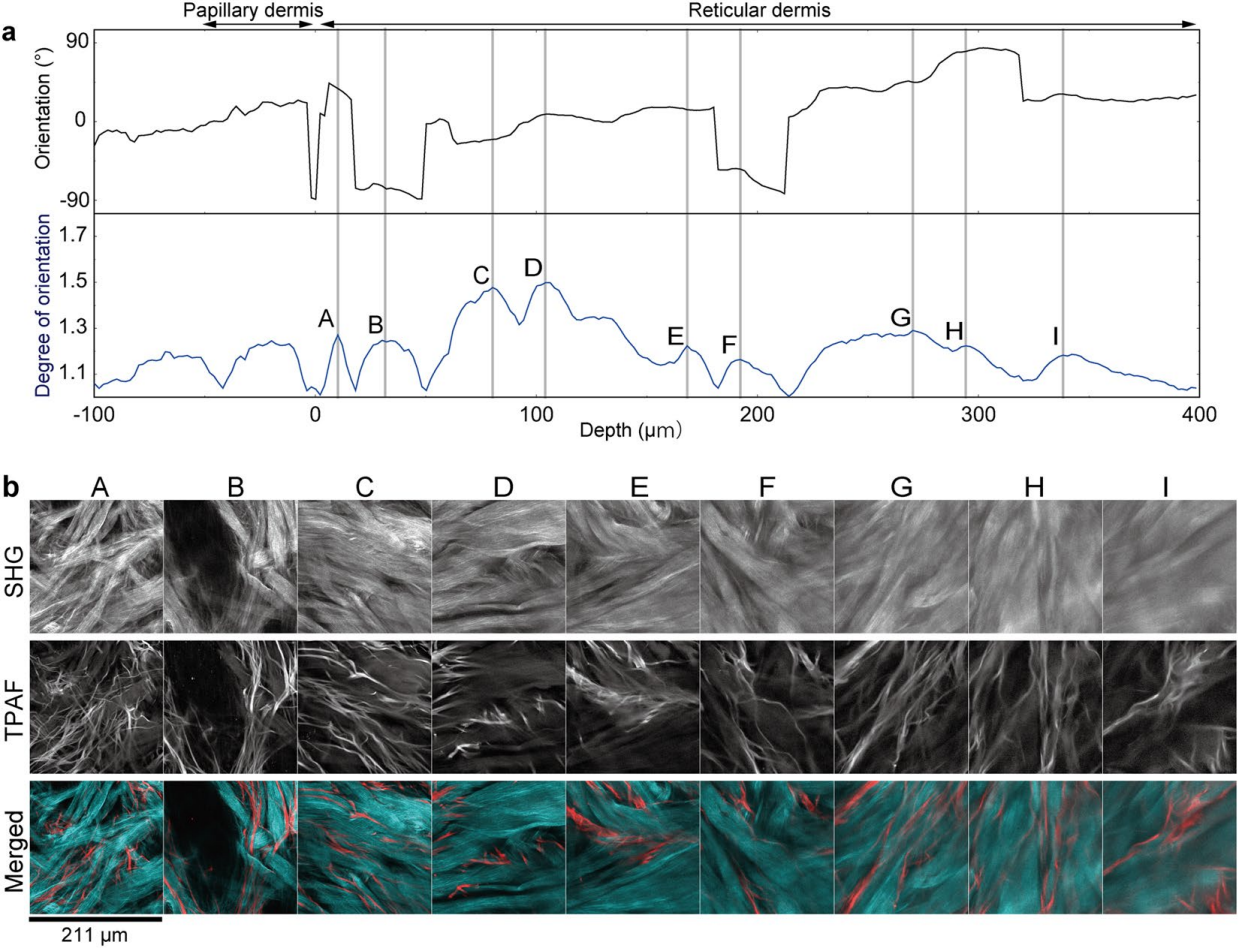
Identification of respective collagen fiber bundles based on peak searching. (**a**) Plot shows changes in the orientation angle (black) and the degree of orientation (blue) by depth, based on stacked second harmonic generation (SHG) images obtained in a 211 × 211 µm area. Assuming the depth of the papillary dermis–reticular dermis boundary to be 0 and the direction from left to right in the image is equivalent to an angle of 0°, and the angle increases in the positive direction in the counter clockwise direction, A–I show representative peaks in the degree of orientation. Note the substantial change in angle between peaks A and B, E and F, and H and I. (**b**) SHG images (collagen fibers), two-photon autofluorescence (TPAF) images (elastic fibers), and merged images at depths corresponding to peaks A–I in **a**. Note the similarities in the direction of collagen and elastic fibers.

We used z-stacked images that were 211 × 211 μm in the x-y plane to evaluate the angle and degree of orientation (mean z-thickness, 375 ± 14 µm for the reticular dermis; n = 6). The orientation angle in SHG images varied by depth; the degree of orientation showed multiple peaks of changes (Fig. 3). The average number of peaks for the degree of orientation was 9 ± 3. The mean interval between peaks for the degree of orientation was comparable for the superficial and deep reticular dermis (30 ± 5 µm vs. 44 ± 15 µm; *p* = 0.07). The collagen fibers in the deep reticular dermis had a larger mean horizontal diameter than in the superficial reticular dermis (47 ± 18 µm vs. 136 ± 14 µm; *p* < 0.01).

**Figure 4:**
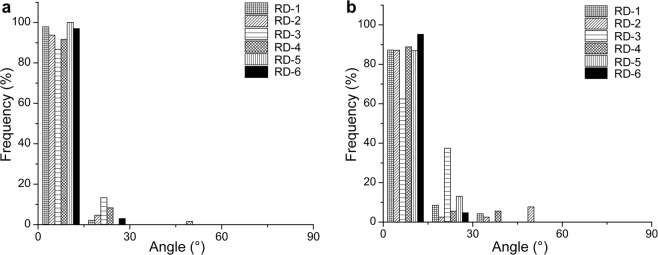
Localized similarities between the direction of collagen and elastic fibers. Histograms show local differences between the orientation angles of collagen and elastic fibers in the superficial reticular dermis (**a**) and the deep reticular dermis (**b**) (x-axis interval, 15°). RD-1 to RD-6 indicate six skin samples.

The mean horizontal diameter of elastic fibers was significantly larger in the deep reticular dermis than in the superficial reticular dermis (4.4 ± 0.6 µm vs. 5.9 ± 0.8 µm; *p* < 0.05). To assess similarities between the distribution of elastic fibers and collagen fiber bundles, we determined the orientation of elastic fibers using TPAF images at the depth used to obtain the peaks for the degree of orientations in SHG images. In the superficial reticular dermis, 94% ± 4% of orientation angles between collagen and elastic fibers, i.e., the angular difference was 0°–15°, compared with 85% ± 10% in the deep reticular dermis (Fig. 4). A high degree of agreement was observed between the localization of collagen and elastic fibers regardless of the layer (see Supplementary Movie 1).

### Characterization of the three-dimensional distribution of collagen fiber bundles in the reticular dermis

**Figure 5:**
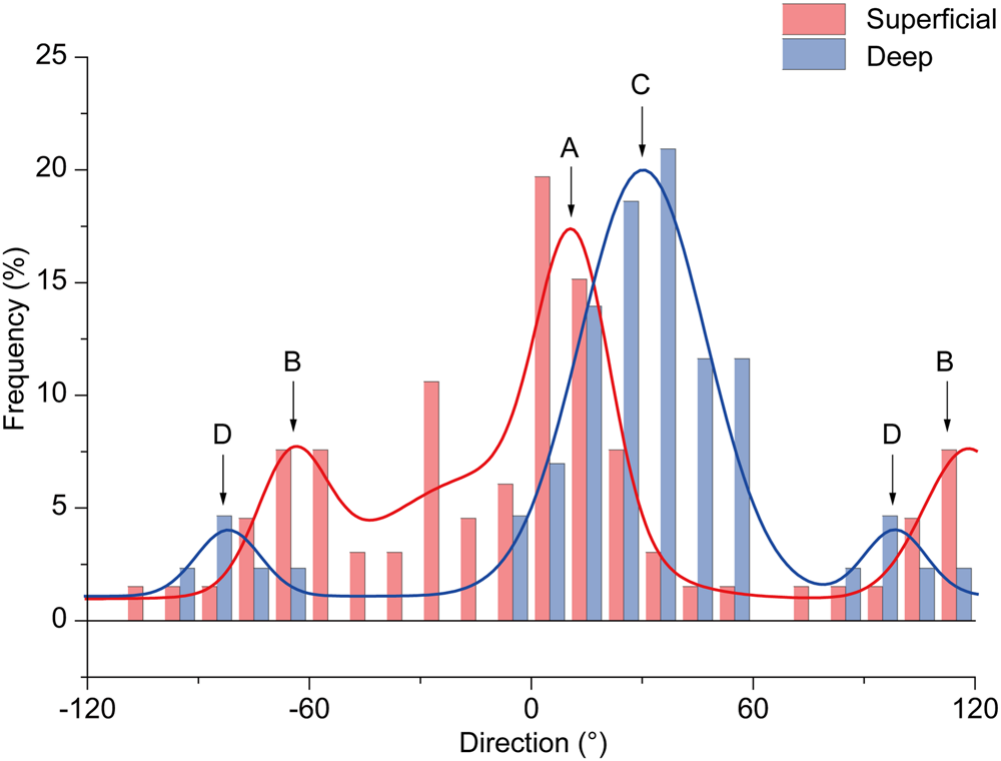
Characterizing the distribution of collagen fibers in the superficial and deep reticular layers. Histogram of the orientation angles of collagen fibers in the superficial reticular dermis (red) and deep reticular dermis (blue) and a representative cumulative curve with Gaussian fitting (RD-6) are shown. Arrows A–D point to the centers of the Gaussian fitting. In this example, collagen fiber bundles have a bimodal distribution, where A and B are the modes with the highest peaks (Mode 1). In contrast, B and D are the modes with the next highest peaks (Mode 2).

A histogram was prepared using data on orientation angles for collagen fiber bundles contained in nine adjacent reticular dermis areas of 211 × 211 μm, i.e., a square region with a total size of 633 × 633 μm. The histogram was used to compare the distribution of the superficial and deep reticular dermis with fitting of the Gaussian function (Fig. 5). Two or three modes of distribution were observed in all six superficial reticular dermis samples; superiorities and inferiorities were found in the peaks, which corresponded to modes (Table 1). In the superficial reticular dermis, the average intermodal angle was 66° (range, 47°–82°). Two or three modes of distribution were also observed in four of six deep reticular dermis samples. The remaining two samples had a unimodal distribution. The average intermodal angle in the deep layer was 60° (range, 46°–69°). For Mode 1, the peak values in the deep reticular dermis in all six samples were higher than those in the superficial reticular dermis, suggesting that collagen fibers are more likely distributed in a single preferential direction in the deeper dermis. For Mode 1, the mean angular difference of the center of modes between the superficial and deep layers was 9° ± 8° (range, 0°–20°). This indicated that the major collagen distribution of the superficial and deep reticular layers was similar.

**Table 1:** Characteristics of the distribution of collagen fiber bundles in the superficial and deep reticular dermis in six samples (RD-1 to RD-6).

Sample	Superficial layer (0 ≤ d < 200 µm)			Deep layer (d ≥ 200 µm)			Angle difference between superficial and deep layers (°)	
	Peak frequency (%)		Intermodal angle (°)	Peak frequency (%)		Intermodal angle (°)		
	Mode 1	Mode 2		Mode 1	Mode 2		Mode 1	Mode 2
RD-1	25	6	80	26	—	—	1	—
RD-2	17	9	47	20	9	60	18	31
RD-3	16	12	59	31	6	65	12	6
RD-4	13	4	56	25	6	46	5	15
RD-5	22	8	82	46	—	—	0	—
RD-6	17	8	72	20	4	69	20	18

Modes with the highest peaks with Gaussian fitting are defined as Mode 1. Modes that have the next highest peaks are defined as Mode 2. d indicates the depth from the papillary dermis–reticular dermis boundary.

## Discussion

Studies on the geometric organization of collagen fibers in the dermis began with the discovery that the mechanical characteristics of human skin have a directional dependence (anisotropy). Langer made an anisotropy map for the whole body, called Langer’s lines^22^. Since anisotropy was observed in skin *ex vivo*^13,15,23,24^, it was thought that anisotropy could be based on the anatomy of the dermis. Preferential distribution of collagen fibers parallel to Langer’s lines was mostly documented in the early literature^15,23^. However, studies using scanning electron microscopy failed to support this distribution^5,6^. Some authors attempted to characterize dermal collagen fiber distribution quantitatively. Brown characterized the distribution by analyzing data on patterns of fiber intersection using electron microscopy images and concluded that it is likely multidirectional^5^. Recently, Ni Annaidh *et al*. evaluated the orientation of collagen fibers in human abdominal skin using serial histological specimens and computerized methods. They found a preferential distribution^25^. Although computerized methods for analyzing fiber orientation are available, it should be noted that the arrangement of collagen fibers varies by the amount of force exerted on the dermis^9,10^. Therefore, methods that standardize the tensile state of the skin during microscopic observation are essential for quantitatively evaluating the geometric organization of the dermis.

Piérard and Lapière observed tissues *ex vivo* in a constant tensile state by attaching a ring to the skin and using scanning electron microscopy^26^. Although their study revealed no preferential orientation of large collagen bundles in samples of adult reticular dermis, they offered a method for maintaining skin samples in near *in vivo* conditions. However, skin tension varies depending on joint position, posing a challenge for standardization. Instead, we employed a method in which the skin was allowed to spontaneously contract, followed by application of biaxial extension. This method does not necessarily recreate the tensile state of the skin *in vivo*, but has an advantage of high reproducibility of the tensile state. Based on the mechanical test data, the degree of extension was restricted to 1.25 to avoid possible injuries to the collagen fibers. Indeed, application of biaxial extension revealed linearization of collagen fibers while conserving the original orientation; thus, the geometric distribution of tissues after biaxial extension was considered to reproduce the geometric organization before extension.

MPM is a powerful tool for characterizing the architecture of collagen-rich organs. Using near-infrared light, a laser beam can penetrate deeper in the skin; however, it is difficult to visualize collagen fibers at depths of ≥100 μm with high resolution using a commonly used Ti:Sapphire laser due to light scattering caused by differences in the reflection coefficients of the epidermis and dermis^21^. In this study, the tissue clearing method was also used to improve the penetration of the laser beam. We used the Clear, Unobstructed Brain Imaging cocktails and Computational analysis (CUBIC) protocol because its applicability to the skin has been established^27^. The CUBIC protocol induces mild swelling in tissues^28^. However, in our preliminary experiment, tissue swelling was ≤5% (data not shown), so the effect of swelling on fiber orientation was assumed to be minimal. Skin samples that underwent biaxial extension were processed using the clearing method; this allowed observation at a depth of approximately 500 μm from the skin surface. This depth is equivalent to ~850 μm in unstretched skin. However, the deepest layer of the reticular dermis (i.e., the layer adjacent to the hypodermis) may not have been analyzed, which is a limitation of this study.

Collagen fiber distribution in multiple directions is one of highlights of this study. In early studies, a lattice-like organizational model of dermal collagen fibers was proposed^15,23^. In this model, the mechanical anisotropy of the skin was explained by collagen fibers arranged in a rhomboidal shape, moving in a manner of a pantograph in the horizontal direction. This model assumes a bimodal distribution of collagen fibers in the human dermis; however, microstructures that support this model have not been observed. Moreover, lattice size was not documented. We observed a 633-μm square-shaped area between hair roots and evaluated the distribution of collagen fiber bundles in this area. Interestingly, all six samples had distributions with two or three modes in the superficial layer. Moreover, superiorities and inferiorities were noted among modes. This result does not agree with models of a simple rhombus architecture or preferential distribution. Rather, it indicates an architecture in which the distribution of a small number of fiber bundles intersects with fiber bundles with the major distribution. In addition, the major distribution of the superficial and deep reticular layers was similar.

Another highlight of this study was the high degree of local similarities observed between the orientation angles of collagen and elastic fibers, which could provide insight into skin elastogenesis. The elastic system of human skin consists of three different types of fibers: oxytalan fibers and elaunin fibers in the papillary dermis and elastic fibers in the reticular dermis^29^. Elastic fibers are composed of an elastin core and surrounding fibrillin microfibrils. Recently, a wide array of fibrillin biding proteins was shown to be associated with fiber assembly^30,31^. Embryologically, there is a discrepancy in timing between the assembly of collagen and elastic fibers. The collagen fiber architecture of the papillary dermis and reticular dermis is established by the second trimester of pregnancy whereas the network of elastic fibers develops in the third trimester^4,32^. This discrepancy suggests that the organization of collagen fibers might be involved in the formation of the elastic fiber network. Recent *in vitro* studies revealed an association between stretching environment and tissue organization^33,34^. Future studies on the generation and remodeling of collagen and elastic fibers may clarify the mechanisms underlying the distributional similarity between fibers.

**Figure 6:**
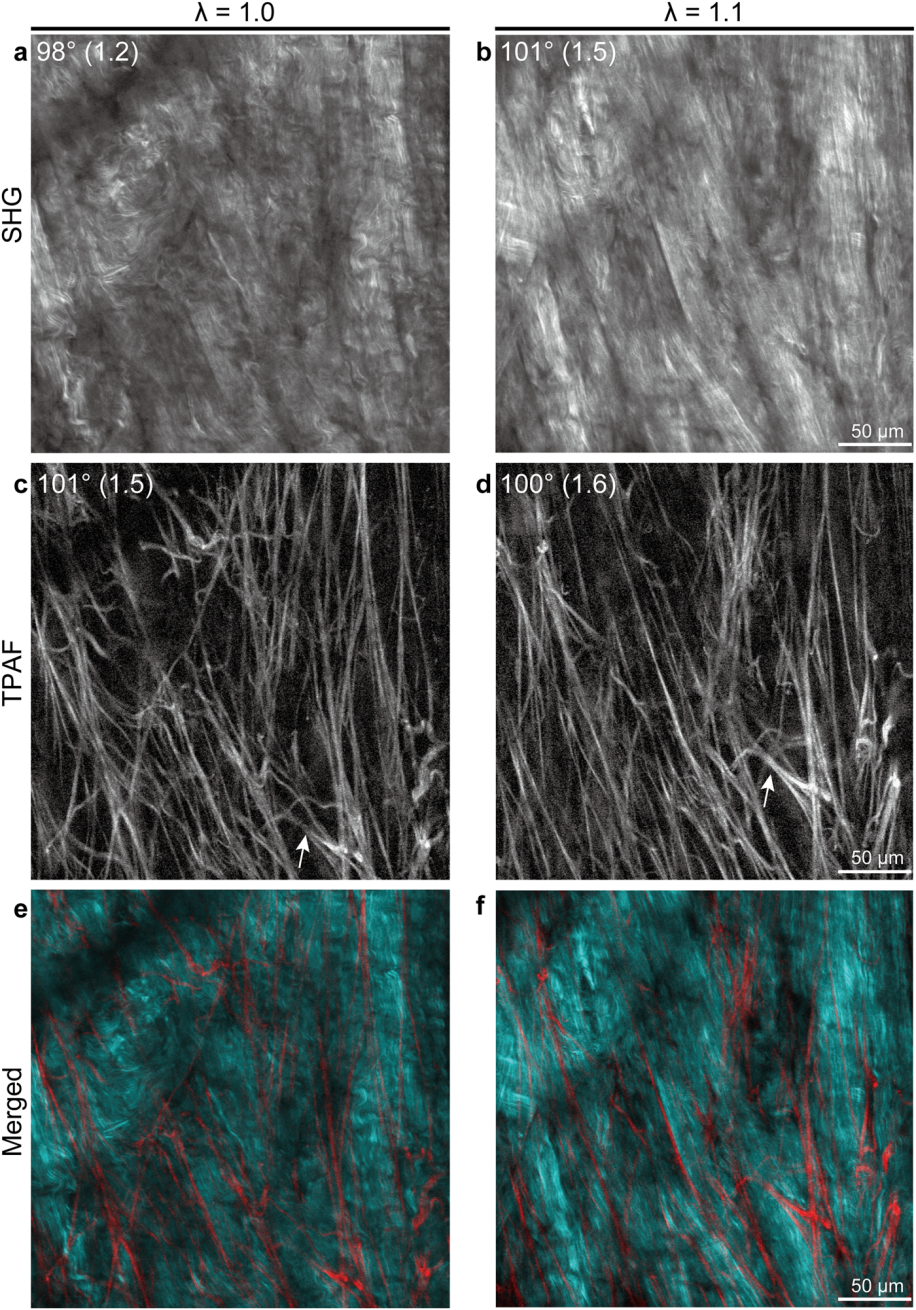
Dynamic multiphoton microscopy images of joint ligament architecture under biaxial tissue extension. Second harmonic generation (SHG) images show architectural changes in collagen fibers (**a**,**b**) whereas two-photon autofluorescence (TPAF) images show architectural changes in elastic fibers (**c**,**d**). λ represents the stretch ratio. Arrows of the same shape indicate the same elastic fiber. The orientation and degree of orientation (parentheses) are shown for each SHG and TPAF image. Note that regularly aligned collagen fibers became more identifiable because they became less wavy, resulting in an increased degree of orientation on extension.

**Figure 7:**
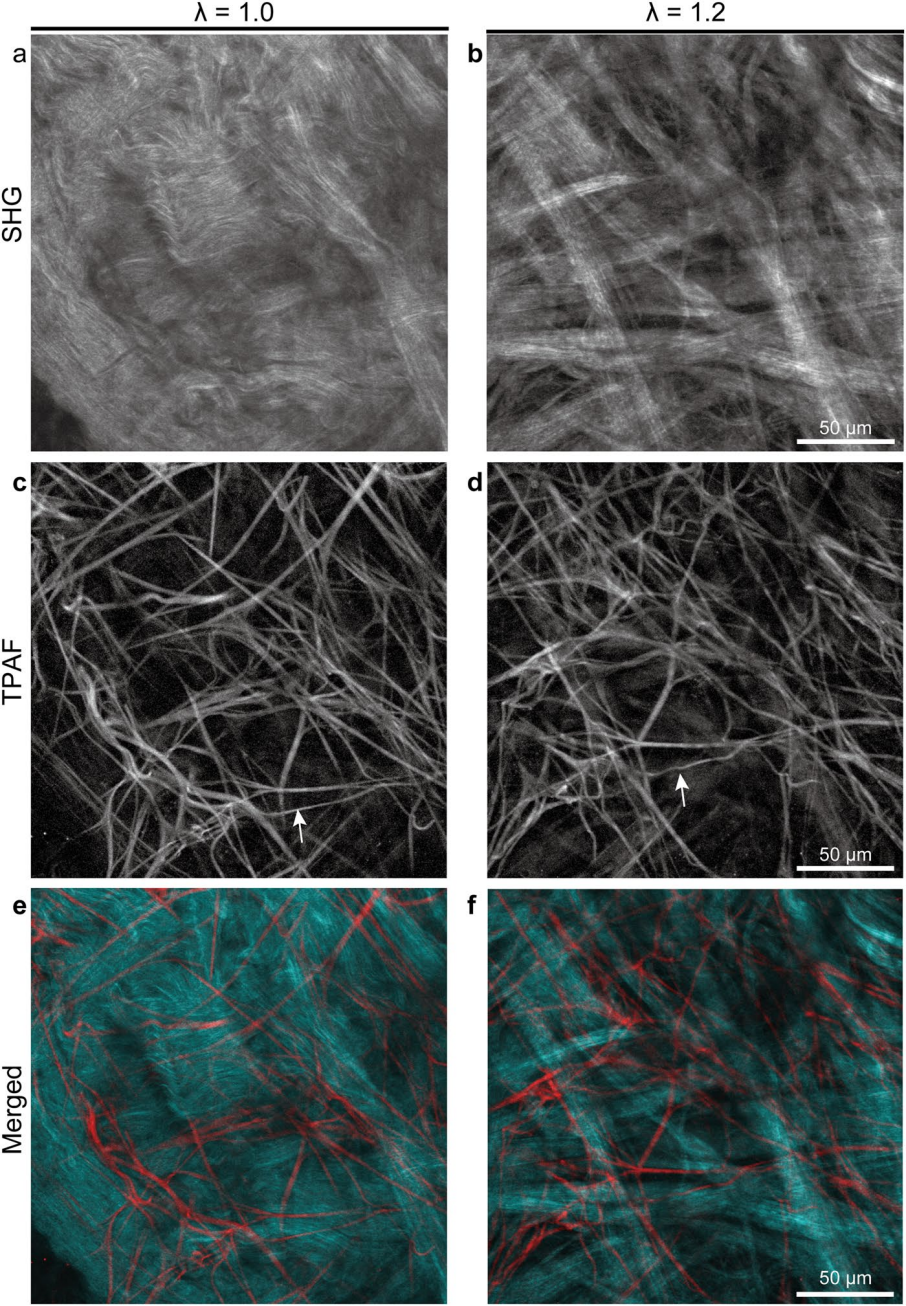
Dynamic multiphoton microscopy images of periadventitial architecture under biaxial tissue extension. Second harmonic generation (SHG) images show architectural changes in collagen fibers (**a**,**b**) whereas two-photon autofluorescence (TPAF) images show architectural changes in elastic fibers (**c**,**d**). λ represents the stretch ratio. Arrows of the same shape indicate the same elastic fiber. Note that collagen architecture changed from a tightly packed appearance to a basket weave-like appearance with extension.

Future research applications of the imaging technique include other connective tissues. We tested the applicability of our technique for various connective tissues, such as scars, tendons, ligaments, and arterial walls. Tendons contain regularly aligned collagen fibers with less deflection; a periodic optical pattern, termed crimp^35^, can be observed. No apparent improvement in fiber visibility could be confirmed with biaxial stretching because collagen fibers were linear in the unstretched condition. Elastic fibers were not observed with TPAF. Scars also contain regularly aligned collagen fibers with small periodic crimps. The degree of orientation increased slightly on extension due to straightening of the fibers (Supplementary Fig. S2). In contrast, the visibility of collagen fibers in ligaments was improved by extension, even though they had a relatively regular distribution because they were moderately wavy (Fig. 6). When they were stretched, the waviness in the fibers was removed, resulting in an increased degree of orientation. The visibility of collagen fibers in the periadventitia was dramatically improved with biaxial extension (Fig. 7). Before extension, individual collagen fibers could not be distinguished due to their tightly packed distribution. On extension, a basket weave-like structure of collagen appeared. We believe that this technique could be useful for analyzing the structure of other flat connective tissues, in particular, tissues in which wavy collagen fibers are densely distributed.

## Conclusions

We visualized the fiber architecture of the reticular dermis of the human skin using a combination of MPM, the tissue clearing method, and biaxial extension. Especially in the superficial reticular dermis, collagen fibers had an architecture in which the distribution of a small number of fiber bundles intersected with fiber bundles with the major distribution. The major collagen distribution in the superficial and deep reticular dermis was similar. Local similarities in the direction of collagen and elastic fibers were observed. The combined use of MPM, the tissue clearing method, and biaxial extension allows for quantitative analysis of the geometric organization of collagen and elastic fibers in the reticular dermis. We believe that the combination of MPM and biaxial extension could be a powerful research tool for studying the fibrous microarchitecture of various connective tissues.

## Methods

A flowchart of evaluation methods is shown in Supplementary Fig. S1. This study was approved by the ethics committee of Kyoto University (approval number, R0700) and conducted in accordance with the Declaration of Helsinki. Written informed consent was obtained from all subjects.

### Sample preparation

Surplus skin was collected by trimming a skin flap during reconstructive surgery involving a free skin flap. Samples used for comparing the visibility of collagen fibers between unstretched and stretched samples were from the abdomen. Skin samples with sufficient size for this test were obtained from the abdomen because a relatively large abdominal flap is frequently excised during reconstructive breast surgery. Donor sites of samples used for mechanical tests, dynamic observation, and full-thickness observation were all on the lateral upper thigh. We did not use abdominal skin samples for these purposes because the donors were middle-aged women, in whom the native dermal architecture could have been altered during pregnancy. The samples consisted of healthy skin without any existing trauma or skin disease. Skin samples were obtained from seven donors (six men and one woman) with a mean age of 65.6 ± 12.7 (range, 36–75) years and a mean body mass index of 21.4 ± 2.7 (range, 17.2–26.6) kg/m^2^. Using a Padgett dermatome, the subcutaneous fat layer was removed. Sheets containing only the epidermal and dermal layers of the skin were prepared. Fresh skin samples for dynamic microscopic observation were moistened using phosphate buffer and used within 12 h of sampling. Samples for the mechanical extension test, biaxial extension, and the clearing process were frozen at −140 °C until use and were warmed to room temperature before use.

### Mechanical extension test

Sheets that contained only the reticular dermis with a thickness of approximately 400 µm derived from full-thickness skin samples were used. Two strips of the same size (15 × 3 mm) were prepared using each sheet, parallel and perpendicular to the direction of body hair, for use in the tensile test. A load-displacement measurement unit (FSA-0.5HK2-20N, IMADA CO., LTD., Aichi, Japan) was used as the extension test device. The initial interval was set at 10 mm, and uniaxial extension was performed at 10 mm/min. Particles of 200 µm were placed at an interval of 5 mm at the center of the sample strips in order to generate strain in the skin. Stress was calculated by dividing the force applied on the skin sample during extension by the initial cross-sectional area to generate a stress-strain curve. Linear approximation was performed for the linear range under high stress, and the value of the x fragment was then measured using this linear approximation.

### Dynamic microscopy

An extension device that applies in-plane biaxial extension was specifically designed. This device comprises a hollow disc with an opening diameter of 7 mm and a donut-shaped pulley with a diameter of 5 mm and a radius of 0.5 mm. The disc moves coaxially along the central axis of the pulley. Tissue sheets mounted on the disc were stretched by the pulley, causing biaxial extension at the center of the tissue sheet. To verify the conservation of fiber distributions in the dermis during extension, an extension device of the same design was installed during MPM assessment and extension was applied to a fresh dermis sheet until collagen fibers became linear. Exploiting the thin morphology of elastic fibers, coordinate data was acquired using imaging software (Kurumi, Kyoto University, Kyoto, Japan). To verify similar expansion of the fiber network due to extension, coordinate values were used to determine the main component vectors. Similarities in the main component vectors before and after extension were evaluated based on cosine similarity.

### Tissue clearing method

A 12 × 12 mm square segment was obtained from a full-thickness skin sample. After stretching tissues in all directions 1.25-fold using the biaxial extension device, samples were immersed and fixed using 4% paraformaldehyde for 24 h in their stretched state. Large full-thickness skin samples were fixed in an unstretched state. To maximize the penetration of the laser beam used in microscopic observation, the tissue clearing (CUBIC) method was used^27,28^. Briefly, tissues were immersed in Reagent-1 at 37 °C for 1 week, which consists of 25 wt% urea (Nacalai Tesque Inc., Kyoto, Japan), 25 wt% N,N,N′,N′-tetrakis(2-hydroxypropyl)ethylenediamine (Tokyo Chemical Industry Co., Ltd., Tokyo, Japan), and 15 wt% polyethylene glycol mono-p-isooctylphenyl ether/Triton X-100 (Nacalai Tesque Inc.)) and then immersed in Reagent-2 for 3 days, which consists of 50 wt% sucrose (Nacalai Tesque Inc.), 25 wt% urea, 10 wt% 2,2′,2′′-nitrilotriethanol (FUJIFILM Wako Pure Chemical Co., Ltd., Tokyo, Japan), and 0.1% (v/v) Triton X-100).

### Image acquisition

The center of each specimen processed using the tissue clearing method was removed using a circular dermal punch of 2.5-mm diameter for microscopic observation. Specimens were placed on a cover glass that was set on the electric x-y stage of a scanning multiphoton laser microscope (FV1000-MPE2, Olympus, Tokyo, Japan) so that images were acquired from the side of the epidermis. After confirming the position of the hair roots, the center of the area between the roots was examined using an objective lens with numerical aperture = 1.05, 30x magnification, and oil immersion (UPLSAPO 30 XS, Olympus). A one-box ultrafast Ti:Sapphire laser (Mai Tai® DeepSee, Spectra-Physics, Santa Clara, CA) with a pulse width of 140 fs and a repetition rate of 80 MHz was used as the light source. The central wavelength and scanning rate (pixel time) were set at 860 nm and 2 μs, respectively. Collagen fibers were detected through a 420–460 nm filter using SHG. Elastic fibers were detected through a 495–540 nm filter using TPAF. The x-y imaging range was set to 211 × 211 µm and 512 × 512 pixels. A total of nine images (adjacent 3 × 3: 633 × 633 µm) was scanned. Laminated image data was made by scanning up to a depth of 500 µm from the skin surface with a z interval of 2 µm. The level of the rete subpapillare was defined as the boundary between the papillary dermis and reticular dermis^36^. The reticular dermis was classified based on the depth (d) from this boundary, where 0 ≤ d < 200 µm corresponds to the superficial reticular dermis and d ≥ 200 µm corresponds to the deep reticular dermis.

### Fourier transform of two-dimensional images and distribution analysis

The direction was assessed using two-dimensional Fourier transformed images^37^. Fourier transformation was performed on each 211 × 211 µm square image. This processing was continuously performed for 251 images in the z direction, with a total of 2,259 images for each sample (nine adjacent regions per sample). Briefly, 8-bit, 512 × 512 pixels, and 211 × 211 µm images were Fourier transformed to obtain power spectrum images. From the power spectrum images, the mean amplitude in each direction was calculated and plotted using polar coordinates. Ellipse approximation was applied on the plot. The minor axis of the ellipse was defined as the orientation angle. The axial ratio was defined as the degree of orientation. If a distinct large bundle of collagen fibers appeared in the z-stacked images, then the degree of orientation increased. When the bundle disappeared, the degree of orientation decreased. Based on this characteristic, the first derivative of the z-depth and the plot of the degree of orientation were used to search for peaks. Peaks with a degree of orientation <1.10 were excluded as unimportant. The orientation angle on TPAF images at the level consistent with that of the degree of orientation peaks on SHG images was used to evaluate the local relationship between the direction of collagen and elastic fibers. A histogram of orientation angles at the identified peaks was prepared to evaluate the distribution of collagen fibers in the 633 × 633 μm area observed. Subsequently, Gaussian model fitting was performed on the histogram. A data analysis program (Origin Pro, OriginLab Corp., Northampton, MA) was used to confirm if the Gaussian model fits the dataset using the chi-squared test. The peak and center (degrees) of the Gaussian model were used as parameters. If there were two or more modes, modes were numbered based on the height of the peaks. The intermodal angle (angular difference between the centers of Modes 1 and 2) was calculated to evaluate whether collagen fibers have a lattice-like organization.

### Statistical analysis

Normally distributed data are presented as means ± standard deviation. The impact of a factor on the variation of data between the two groups was analyzed using the paired *t*-test. A *p* value of < 0.05 was considered significant.

## Supplementary information

Supplementary movie 1

Supplementary materials

## Data Availability

The datasets generated during and/or analyzed during the current study are available from the corresponding author on reasonable request.
