# Supplementary material for: Combined multiphoton imaging and biaxial tissue extension for quantitative analysis of geometric fiber organization in human reticular dermis
Source: Sci Rep. 2019 Jul 23;9:10644. doi: 10.1038/s41598-019-47213-5 (PMC6650477; doi:10.1038/s41598-019-47213-5)

**Supplementary information file**

**Combined multiphoton imaging and biaxial tissue extension for quantitative analysis of geometric fiber organization in human reticular dermis**

Maho Ueda^1^, Susumu Saito*^1^, Teruasa Murata^2^, Tomoko Hirano^2^, Ryoma Bise^3^, Kenji Kabashima^2^, Shigehiko Suzuki^1^.

*^1^Department of Plastic and Reconstructive Surgery, Kyoto University, Kyoto, Japan*

*^2^Department of Dermatology, Kyoto University, Kyoto, Japan*

*^3^Department of Advanced Information Technology, Kyushu University, Fukuoka, Japan*

***Corresponding author**

Susumu Saito

Yoshida-Konoe-cho, Sakyo-ku

Kyoto, Japan

Postal code: 606-8501

Telephone number: +81-75-751-3613

Fax number: +81-75-751-4340

E-mail address: [susumus@kuhp.kyoto-u.ac.jp](mailto:susumus@kuhp.kyoto-u.ac.jp)

**Supplementary Figure S1.** Flowchart for sample processing and analysis. The dotted lines indicate feedback.

**Supplementary Figure S2.** Second harmonic generation images of human hypertrophic scar tissue in unstretched (left) and stretched (right) conditions. λ represents the stretch ratio. Note that crimps observed in the unstretched fibers are removed by extension.

**Supplementary movie 1.** Merged second harmonic generation (cyan) and two-photon autofluorescence (red) images of the reticular dermis in a biaxially extended human skin sample (633 × 633 μm square area).

**Supplementary Figure S1**

Analysis of orientation for each 211 × 211 µm image using a Fourier transform method

Full-thickness microscopic scans (2,259 images with a 633 × 633 × 500 µm region)

Tissue clearing (CUBIC)

Biaxial extension (1.25-fold)

Sampling

Dynamic microscopic observation of dermal sheets under biaxial extension

Mechanical testing

Evaluation of local differences between the orientation angles of collagen and elastic fibers

Evaluation of collagen fiber distribution in the reticular dermis using compiled orientation data

**Supplementary Figure S2**


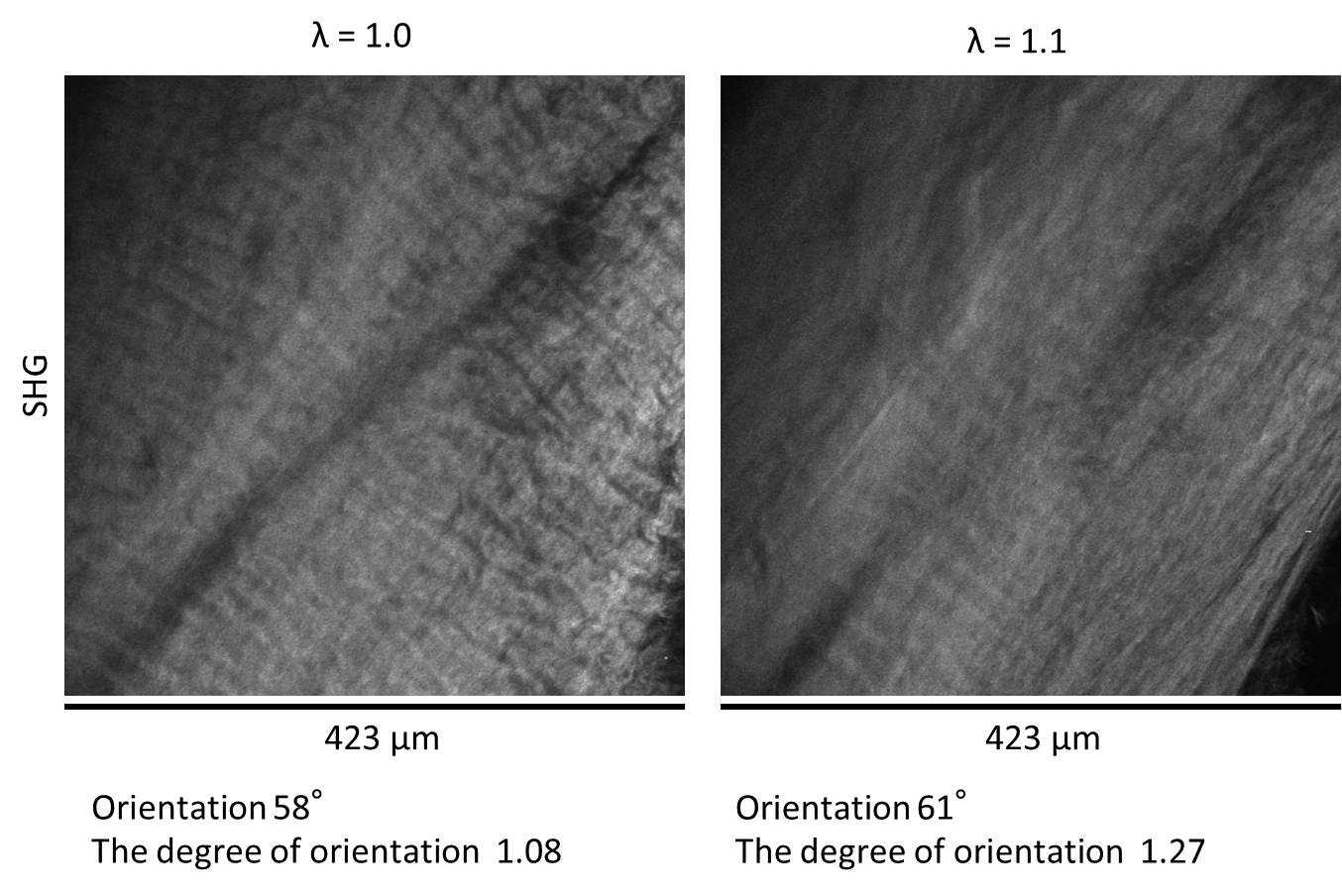

Supplement: Supplementary file 2 — Supplementary materials [file 41598_2019_47213_MOESM2_ESM.docx]
